# Reduced intraepidermal nerve fibre density, glial activation, and sensory changes in HIV type-1 Tat-expressing female mice: involvement of Tat during early stages of HIV-associated painful sensory neuropathy

**DOI:** 10.1097/PR9.0000000000000654

**Published:** 2018-05-14

**Authors:** Rachel Wodarski, Deniz Bagdas, Jason J. Paris, Tim Pheby, Wisam Toma, Ruqiang Xu, M. Imad Damaj, Pamela E. Knapp, Andrew S.C. Rice, Kurt F. Hauser

**Affiliations:** a Pain Research Group, Department of Surgery and Cancer, Imperial College, Chelsea and Westminster Hospital Campus, London, United Kingdom; b Department of Pharmacology and Toxicology, Virginia Commonwealth University, Richmond, VA, USA; c Department of BioMolecular Sciences, University of Mississippi, University, MS, USA; d Department of Anatomy and Neurobiology, Virginia Commonwealth University, Richmond, VA, USA; e School of Life Sciences, Zhengzhou University, Zhengzhou, Henan, China

**Keywords:** HIV-1, Transactivator of transcription, Viral protein, Sensory neuropathy, Rodent behaviour, Digital PCR

## Abstract

**Introduction:**

HIV infection is associated with chronic pain states, including sensory neuropathy, which affects greater than 40% of patients.

**Objectives and Methods:**

To determine the impact of HIV-Tat induction on nociceptive behaviour in female mice conditionally expressing HIV Tat_1-86_ protein through a doxycycline (DOX)-driven glial fibrillary acidic protein promoter, intraepidermal nerve fibre density and immune cell activation in the dorsal root ganglion (DRG) and spinal cord were assessed by immunohistochemistry. Mice were assessed for mechanical and thermal sensitivity for 9 weeks using von-Frey and Hargreaves tests.

**Results:**

Intraepidermal nerve fibre density was significantly reduced after 6 weeks of Tat induction, similar to sensory neuropathy seen in clinical HIV infection. Tat induction through DOX caused a significant reduction in paw withdrawal thresholds in a time-dependent manner starting the 4th week after Tat induction. No changes in paw withdrawal latencies were seen in Tat(−) control mice lacking the *tat* transgene. Although reductions in paw withdrawal thresholds increased throughout the study, no significant change in spontaneous motor activity was observed. Spinal cord (cervical and lumbar), DRG, and hind paw skin were collected at 8 days and 6 weeks after Tat induction. HIV-Tat mRNA expression was significantly increased in lumbar DRG and skin samples 8 days after DOX treatment. Tat induced a significant increase in the number of Iba-1 positive cells at 6 weeks, but not after 8 days, of exposure. No differences in glial fibrillary acidic protein immunoreactivity were observed.

**Conclusion:**

These results suggest that Tat protein contributes to painful HIV-related sensory neuropathy during the initial stages of the pathogenesis.

## Abstract 2

Supplemental Digital Content is Available in the Text.

## 1. Introduction

With a prevalence of 40% to 50%, HIV-associated sensory neuropathy (HIV-SN)—a distal symmetrical, sensory polyneuropathy, characterised by a “dying back” pattern of axonal degeneration—is one of the most frequent neurological complications in people living with HIV.^17,64^ Neuropathic pain and resulting comorbidities are common in HIV-SN and significantly contribute to the disease state of patients with HIV.^54,55^ HIV-SN can originate from 2 clinically indistinguishable neuropathies, an antiretroviral toxic neuropathy and/or a distal sensory polyneuropathy.^33^ Initially, exposure to neurotoxic dideoxynucleoside reverse transcriptase inhibitors was believed to be the main underlying cause of HIV-SN^12,29^; however, after cessation in dideoxynucleoside reverse transcriptase inhibitor use, the prevalence of HIV-SN remains high, even in patients who have not been exposed to antiretroviral drugs of known neurotoxicity.^17,33,64^ This suggests a more prominent role of the virus itself in HIV-SN pathogenesis.

Given that neurons are not directly targeted by the virus,^75^ infected macrophages and glia (primarily microglia and, to a lesser extent, astrocytes) are likely involved in the HIV-SN etiology. Indeed, viral proteins produced by infected cells can exhibit neurotoxic properties in uninfected neurons. Neurotoxicity has been described for 5 of the 9 major HIV proteins, but with respect to HIV-SN and concomitant neuropathic pain, research has focused primarily on the envelope glycoprotein 120 (gp120), which mediates neurotoxicity directly and indirectly, through activation of immune/glial cells resulting in the release of inflammatory mediators.^4,33,49,70,71^

A viral protein that could equally contribute to the pathogenesis of HIV-SN is HIV-1 transactivator of transcription (Tat), which has been extensively studied regarding another HIV-related neurological complication—HIV-associated neurocognitive disorders. In vitro, Tat is reportedly directly neurotoxic by binding of the lipoprotein receptor-related protein, which results in an exaggerated, excitotoxic response of *N*-methyl-d-aspartate receptors to glutamate.^25,57^ Tat-induced hyperexcitability of neurons may be further facilitated through decreased glutamate uptake by astrocytes^82^ and through activation of immune and glial cells and consequential release of inflammatory cytokines.^16,43,62,66,80^ Neuronal damage and glial cell activation have also been demonstrated in vivo in Tat-expressing transgenic mice.^8,24,36,46^

Tat expression has largely been validated in the central nervous system (CNS) of Tat-overexpressing mice and patients with HIV.^19,24,74,76^ However, Tat can also be measured in the cerebrospinal fluid and in the peripheral blood samples of patients.^3,6,47,79^ The importance of Tat outside the CNS in HIV pathogenesis is highlighted by an inverse correlation of plasma Tat antibody levels with disease progression.^6,58^ High antibody levels may sequester/neutralise Tat function and therefore protect against its toxic properties. Notably, the presence of Tat is not attenuated by reduced viral load, even in HIV patients with good viral control receiving antiretroviral treatment.^32,47^ Therefore, persistent Tat secretion in chronic HIV infection may contribute to the axonal degeneration in HIV-SN.

We hypothesize that HIV-Tat is an important factor in HIV-SN pathogenesis and investigated whether epidermal nerve fibre loss and changes in sensory thresholds, both characteristics of clinical painful HIV-SN, were present in a transgenic mouse model of inducible HIV-1 Tat expression.

## 2. Methods

### 2.1. Ethical statement

All in vivo procedures in the United States were preapproved by the Institutional Animal Care and Use Committee at Virginia Commonwealth University, and experiments were conducted in accordance with ethical guidelines defined by the National Institutes of Health (NIH Publication No. 85-23). Studies were conducted based on Good Laboratory Practice guidelines^44,60^ (Table 1).

**Table 1:**
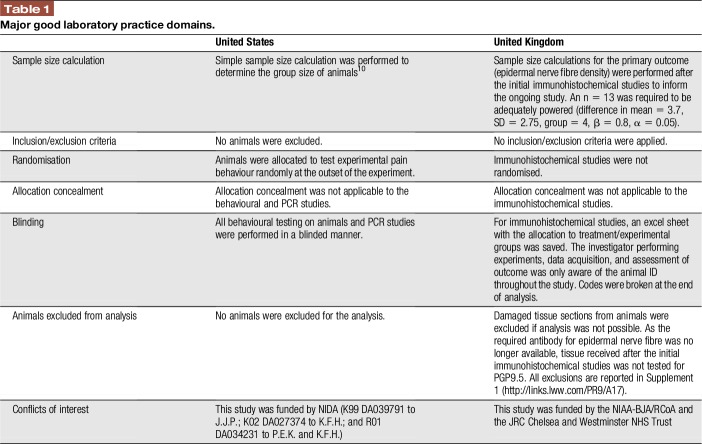
Major good laboratory practice domains.

### 2.2. Animals and environmental conditions

For all experiments, female mice (8–10 weeks; 25–30 g at the start of the experiment) were used. Mice were housed in groups of 3 in a 21°C, humidity-controlled, Association for Assessment and Accreditation of Laboratory Animal Care (AALAC)-accredited animal care facility. Animal rooms were on a 12-hour light/dark cycle (lights on at 7:00 am), and food and water were freely available.

#### 2.2.1. HIV Tat_1-86_ transgenic mice

HIV Tat_1-86_ expression was induced through a tetracycline (*tet*) “on” system in mice on a C3H × C57BL/6J background.^8^ Briefly, a reverse *tet* transactivator (rtTA), under control of a glial fibrillary acidic protein (GFAP) promotor, was activated in the presence of doxycycline (DOX; 6 g/kg in chow; Harlan, IN). Tat(+) mice expressed GFAP-driven Tat_1-86_, whereas Tat(−) mice only expressed the rtTA transcription factor. To account for potential nonspecific effects of DOX treatment, both Tat(+) and Tat(−) mice were maintained on a DOX chow diet. Details of the mice used in the experiments are provided (Supplemental Table 1, available at http://links.lww.com/PR9/A17).

### 2.3. Behavioural testing

All behavioural experiments were performed during the light cycle and with the observer unaware of the genotype of the animals.

#### 2.3.1. Mechanical hypersensitivity

Mechanical withdrawal thresholds were determined, with slight modifications, as previously described.^11^ Mice were placed in a Plexiglas cage on a mesh metal flooring and allowed to acclimatise for 30 minutes before testing. Withdrawal thresholds were measured by applying a series of calibrated von-Frey filaments (Stoelting, Wood Dale, IL; logarithmically incremental force from 2.83 to 5.88 expressed in dsLog 10 of [10 pound force in milligram]) to the hind paw. Using a modified up-down method,^5^ in the absence of a paw withdrawal response (paw withdrawn, licking, or shaking) to the initially selected filament, a thicker filament corresponding to a stronger stimulus was presented. Once a paw withdrawal occurred, the next weaker stimulus was chosen. Each hair was presented vertically against the paw, with sufficient force to cause slight bending, and held for 2 to 3 seconds. A stimulation of the same intensity was applied 3 times at intervals of a few seconds. The mechanical withdrawal threshold was expressed as Log10 of (10 pound force in milligram).

#### 2.3.2. Thermal hypersensitivity

Thermal withdrawal latencies were measured using the Hargreaves test as previously described.^5^ For this, mice were placed in clear plastic chambers (7 × 9 × 10 cm) on an elevated surface and allowed to acclimatise to their environment before testing. The radiant heat source was aimed at the plantar surface of each hind paw in the area immediately proximal to the toes. The paw withdrawal latency (PWL) was defined as the time from the onset of radiant heat until a withdrawal of the hind paw occurred. A 20-second cutoff time was used to avoid tissue injury and sensitisation. Three measures of PWL were taken and averaged for each hind paw.

#### 2.3.3. Locomotor performance

Locomotor activity was measured by placing mice into individual Omnitech (Columbus, OH) photocell activity cages (28 × 16.5 cm). Interruptions of the photocell beams (2 banks of 8 cells each) were recorded for 30 minutes. Data were expressed as the number of photocell interruptions.

### 2.4. Tissue collection

Tat mRNA levels were analysed in tissue obtained from Tat(+) and Tat(−) mice after 8 days of Tat induction. For tissue used to quantify Tat mRNA levels, mice were anaesthetised with 4% isoflurane through inhalation before killing by dislocation of the neck. Spinal cord (cervical and lumbar), dorsal root ganglion (DRG), and skin samples were dissected and snap-frozen in liquid nitrogen. Samples were then stored at −80°C until further use.

Histological analyses were performed on tissue obtained from Tat(+) and Tat(−) mice after 8 days or 6 weeks of Tat induction. For tissue used for immunohistochemical studies, mice were anaesthetised with 4% isoflurane through inhalation and thoracotomised before transcardiac perfusion with 4% paraformaldehyde. Skin (dorsal hind paw) and spinal columns (including spinal cords and DRG) were collected and postfixed overnight in 4% paraformaldehyde before transfer to phosphate-buffered saline (PBS). Samples were then shipped in PBS to the United Kingdom and stored at 4°C until further processing.

### 2.5. Detection and quantification of Tat mRNA

#### 2.5.1. Quantitative real-time PCR

Total RNA was isolated from tissue samples of DRG and lumbosacral spinal cord using the miRNeasy Mini Kit (Qiagen, Germantown, MD). After DNase treatment of RNA, reverse transcription was conducted using the High Capacity cDNA Reverse Transcription Kit (Applied Biosystems, Foster City, CA). Real-time PCR reactions were performed in a total volume of 20 μL containing SensiMixTM SYBR qPCR reagents (Bioline USA, Taunton, MA) using a Corbett Rotor-Gene 6000 real-time PCR system (Qiagen) and previously described primers.^19^ PCR conditions consisted of an initial hold step at 95°C for 10 minutes followed by 40 amplification cycles of 95°C for 5 seconds, 58°C for 10 seconds, and 72°C for 15 seconds. Real-time data were collected during the extension step of each cycle. Melting curve analyses were performed at the end of the reaction between 62°C and 95°C to assess the quality of final PCR products. Three independent quantitative real-time PCR experiments were performed for each sample. Quantitative real-time PCR data were calculated as relative expression levels by normalisation against β-actin mRNA using the 2^−ΔΔCt^ method.

#### 2.5.2. Digital PCR

Total RNA was extracted from skin tissue samples, DNase I was used to remove DNA contamination, and RNA reverse-transcribed to generate cDNAs as previously reported.^19^ Digital PCR (dPCR) assays were performed using a QX200TM Droplet DigitalTM PCR System (Bio-Rad, Hercules, CA) following the manufacturer's instruction. Briefly, the dPCR reaction mixture was prepared in a final volume of 20 μL, composed of 11 μL of QX200 ddPCR EvaGreen Supermix (Bio-Rad), 4.4 μL of cDNA template (or 4.4 μL of nuclease-free water for nontemplate controls), 0.2 μL each of forward and reverse primer (final concentration of 100 nM), and 4.2 μL of nuclease-free water. The cDNA templates used in the reaction for β-actin (reference control) and Tat mRNA amplification were diluted 100-fold and 5-fold, respectively. The primers were designed to specifically detect Tat mRNA sequences within our transgenic mice as previously reported.^19^ The 20 μL reaction mixture was loaded into each sample well of an 8-channel disposable cartridge and 60 μL of droplet generation oil was used in each channel of the QX200 droplet generator. Water-in-oil droplets were generated and transferred to 96-well PCR plates. The plates were heat sealed with foil and placed in a T100 PCR cycler (Bio-Rad). After an initial denaturation step (95°C, 5 minutes), the reaction underwent 40 amplification cycles (95°C for 30 seconds and 60°C for 1 minute). After cycling, each sample was incubated at 90°C for 5 minutes, cooled to 4°C, and the droplets in each well were analysed using a QX200 droplet reader (Bio-Rad). Data acquisition and analysis were performed using QuantaSoft analysis software (Bio-Rad). Positive droplets, containing amplification products, were discriminated from negative droplets (lacking amplification products) by applying a fluorescence amplitude threshold. The concentration of the template was calculated based on the number of template copies in the reaction. HIV-1 Tat mRNA-relative expression levels were calculated using the 2^−ΔΔCt^ method as previously reported^14^ and normalized against β-actin.

### 2.6. Immunohistochemistry

For immunohistochemical studies, spinal columns were dissected and the lumbar part of the spinal cord and DRG from the same level were collected. Neuronal tissue and skin samples were cryoprotected by placing them into a 15% sucrose solution in 0.1 M phosphate buffer (15% wt/vol sucrose [BDH, London, United Kingdom], 3.12 g/L NaH_2_PO_4_·2H_2_O [Sigma, Welwyn Garden City, United Kingdom], and 28.65 g/L Na_2_HPO_4_·12H_2_O [Sigma]) for 24 hours at 4°C. Samples were then transferred to a 30% sucrose solution in 0.1 M phosphate buffer for a further 24 hours before tissue was embedded in optimal cutting temperature compound. Fourteen micrometer (skin and spinal cord) and 7 μm (DRG) sections were sectioned on a cryostat and thaw mounted onto glass slides.

For immunohistochemistry, slide-mounted tissue sections were blocked with 10% goat serum (Millipore, Hertfordshire, United Kingdom) in PBS containing 0.2% Triton-X (Sigma) and 0.1% sodium azide (Sigma) (PBS-X). Slides were then incubated overnight at room temperature with primary antibody in PBS-X followed by the appropriate secondary antibody for 2 hours (Table 2). At the end of the protocol, slides were coverslipped with mounting medium (VectaShield, Peterborough, United Kingdom).

**Table 2:**
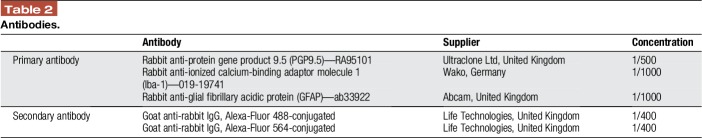
Antibodies.

### 2.7. Visualisation and quantification of immunoreactivity

Slides were visualised under a Leica DMR microscope (Leica, Milton Keynes, United Kingdom), and analysis was performed using ImageJ software (National Institute of Health). Visualisation and analysis was performed blinded to genotype and/or treatment group.

For assessment of epidermal nerve fibres, pictures were taken from 4 to 5 sections per animal (×40 objective) that were picked at random. EFNS guidelines to diagnose peripheral neuropathy using skin biopsies in humans were followed, only counting single fibres crossing the dermal–epidermal junction without counting secondary branching.^40,42^ Epidermal nerve fibre density was presented as fibres per millimeter of skin. For assessment of immunoreactivity in the DRG and spinal cord, pictures were taken from 3 to 4 sections per animals (×20 objective). Only sections from lumbar region L4 to L6 were chosen. Positive immunoreactivity for Iba-1 and GFAP was established by setting the minimum threshold above background values. Background staining was determined by immunoreactivity (arbitrary unit) within neuronal cells that were identified through their clear morphology. Data were represented as percentage of positively stained area across the entire section.

### 2.8. Study design

The primary outcome of the study was to measure the effect of short-term and long-term Tat expression on epidermal nerve fibre density in female mice. Although we are aware of the predominant use of male animals in preclinical studies, we opted for female subjects, as about 52% of people who are currently living with HIV are women.^1^ Furthermore, demographics in clinical studies show no significant difference between HIV patients with and without HIV-SN in terms of sex.^54,64^

Epidermal nerve fibre density was measured in Tat(+) and Tat(−) mice exposed to DOX for 8 days and 6 weeks to induce short-term and long-term Tat expression (n = 10–11/group). The secondary outcome was to identify the effect of Tat expression on neuronal and glial cells in these animals. In a separate group of animals, the effect of Tat expression on sensory thresholds and locomotor activity was assessed. For this, baseline responses were measured for Tat(+) and Tat(−) mice before the animals were put on a DOX diet. Mechanical withdrawal thresholds were then measured weekly and thermal withdrawal thresholds were measured every 2 weeks over a period of 9 weeks (n = 10/group). In addition, animal weight was assessed every week and locomotor activity was measured after 9 weeks of DOX exposure.

### 2.9. Statistics

For Tat mRNA studies and histological studies, data are presented as dot and box plots. Diamonds represent single animal values, whereas the box plot shows mean, median, interquartile range, and SD. Data were analysed by Student independent *t*-tests (comparison of 2 groups) or 1-way analysis of variance (comparison of multiple groups) followed by Bonferroni post hoc analysis. The nonparametric Mann–Whitney and Kruskal–Wallis tests were used where appropriate (SigmaStat 3.5; SysTat Software Inc, Erkrath, Germany). For behavioural studies, data were analysed using GraphPad software, version 6.0 (GraphPad Software, Inc, La Jolla, CA) and are expressed as the mean ± SEM. Statistical analyses were conducted using 2-way repeated-measures analysis of variance, followed by the Sidak post-hoc test to determine group differences. Student independent *t*-tests were used was used for simple 2-group comparisons. Level of significance was set at *P* < 0.05.

## 3. Results

### 3.1. Tat mRNA expression in Tat(+) mice

**Figure 1.**
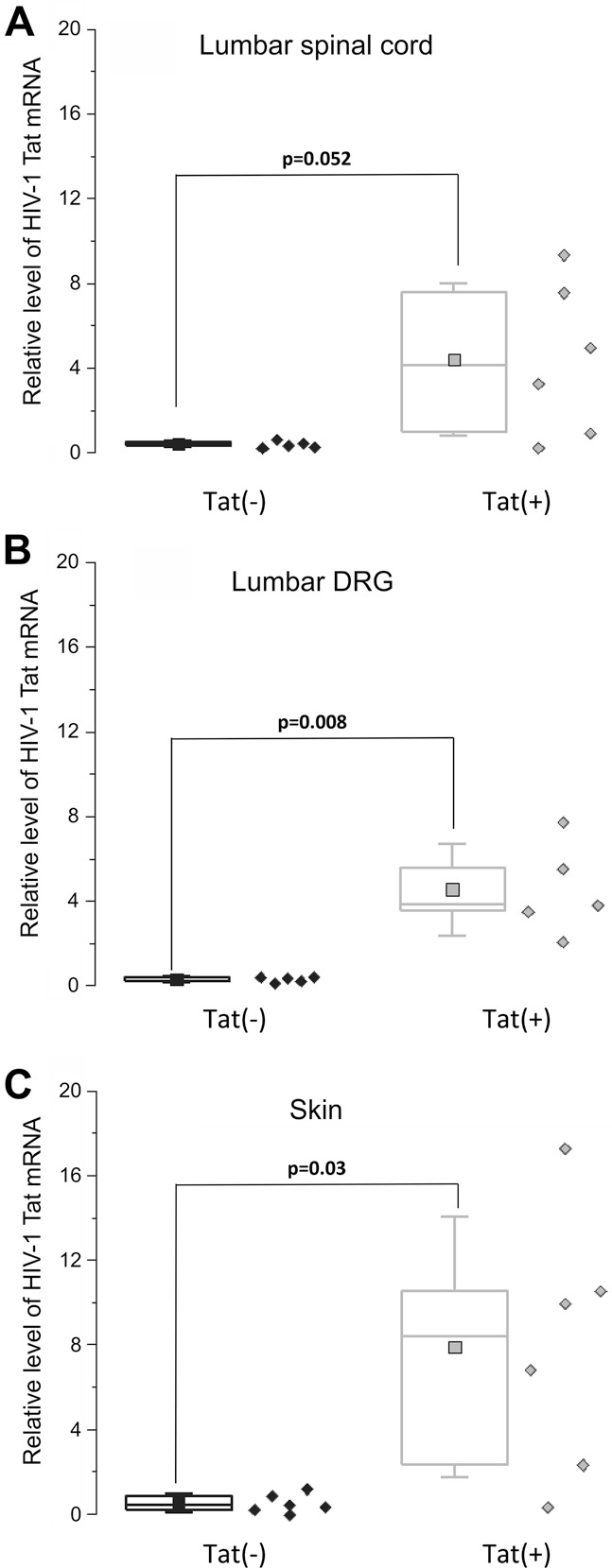
Relative HIV-1 Tat mRNA expression in skin, DRG, and spinal cord of Tat(−) and Tat(+) mice. (A) Relative level of HIV-1 Tat mRNA in the lumbar spinal cord. (B) Relative level of HIV-1 Tat mRNA in lumbar DRG. (C) Relative level of HIV-1 Tat mRNA in skin. Data are shown as box and dot plots. The box represents the interquartile range, with the line representing the median and the square representing the mean. Whiskers show the SD. Diamonds represent individual values for each animal. Data were analysed by a Mann–Whitney test. The *P* value in the upper left corner represents the overall group difference. DRG, dorsal root ganglion.

Tat expression increased 11.3-fold in the lumbar spinal cord of Tat(+) mice, a level that approached, but was not, significant (*P* = 0.052) (Fig. 1A). By contrast, HIV-1 Tat mRNA expression was significantly increased in lumbar DRG and skin of the hind leg 8 days after DOX treatment in Tat(+) mice as compared to Tat(−) mice (*P* = 0.008 and *P* = 0.03 for DRG and skin, respectively) (Fig. 1B, C).

### 3.2. Loss of epidermal nerve fibres in Tat(+) mice

**Figure 2.**
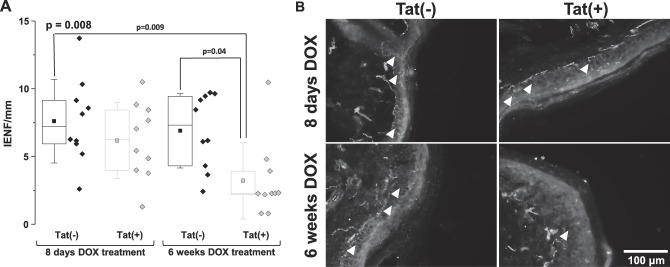
Reduced epidermal nerve fibre density in Tat(+) mice. (A) Intraepidermal nerve fibre density in Tat(−) and Tat(+) mice. Data are shown as box and dot plots with diamonds representing individual animal values. The box represents the interquartile range, with the line representing the median and the square representing the mean. Whiskers show the SD. Data were analysed by a 1-way ANOVA (the Bonferroni post-hoc test). The *P* value in the upper left corner represents the overall group difference. (B) Immunoreactivity to PGP9.5 in Tat(−) and Tat(+) mice. Arrowheads in B indicate individual fibres. ANOVA, analysis of variance; IENF, intraepidermal nerve fibre.

There was no reduction in nerve fibre density observed 8 days after DOX treatment (Fig. 2). By contrast, epidermal nerve fibre density was significantly reduced in Tat(+) as compared to Tat(−) mice after 6 weeks of DOX treatment (*P* = 0.04) (Fig. 2).

### 3.3. No macrophage and satellite cell activation in the dorsal root ganglia of Tat(+) mice

**Figure 3.**
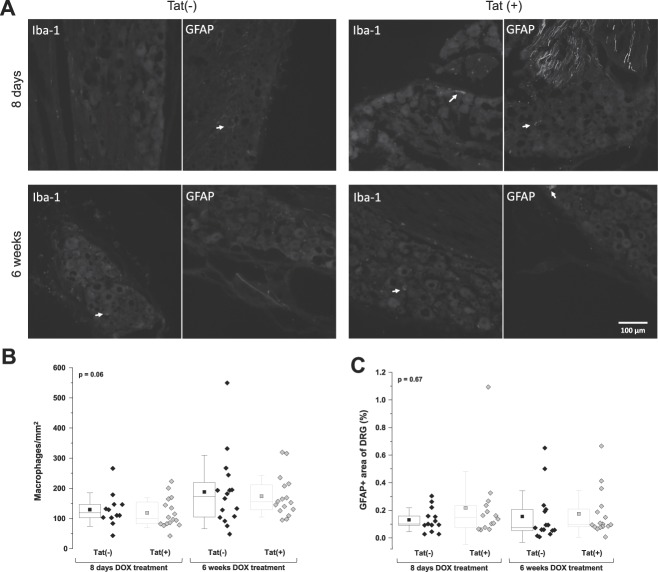
No measurable macrophage and satellite cell activation in the DRG of Tat(+) mice. (A) Immunoreactivity to Iba-1 and GFAP in DRG sections of Tat(−) and Tat(+) mice 8 days and 6 weeks after DOX treatment. Arrows in A indicate Iba-1 or GFAP immunoreactive cells and/or their processes. (B) Number of macrophages per mm^2^ in Tat(−) and Tat(+) mice. Data are shown as box and dot plots with diamonds representing single animal values. The box represents the interquartile range, with the line representing the median and the square representing the mean. Whiskers show the SD. Data were analysed by 1-way ANOVA (not significant). The *P* value in the upper left corner represents the overall group difference. (C) Percent of area with positive immunoreactivity to GFAP in Tat(−) and Tat(+) mice. Data are shown as box and dot plots with diamonds representing single animal values. The box represents the interquartile range, with the line representing the median and the square representing the mean. Whiskers show the SD. Data were analysed by a 1-way ANOVA (NS). The *P* value in the upper left corner represents the overall group difference. ANOVA, analysis of variance; DOX, doxycycline; DRG, dorsal root ganglion; GFAP, glial fibrillary acidic protein.

There was no significant difference between Tat(+) and Tat(−) mice after 8 days or 6 weeks of DOX treatment in macrophage cell number (*P* = 0.06; Fig. 3A, B) or GFAP immunoreactivity (*P* = 0.67; Fig. 3A, C).

### 3.4. Microglial activation, but no astroglial activation, in the spinal cord of Tat(+) mice

**Figure 4.**
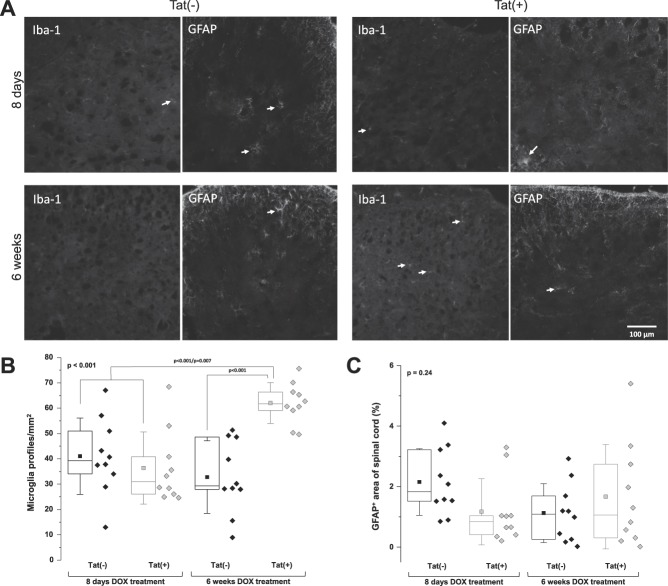
Microglial, but not astroglial, activation in the dorsal spinal cord of Tat(+) mice. (A) Immunoreactivity to Iba-1 and GFAP in spinal cord sections of Tat(−) and Tat(+) mice at 8 days and 6 weeks after DOX treatment. Arrows in A indicate Iba-1 or GFAP immunoreactive cells and/or their processes. (B) Number of microglial profiles per mm^2^ in Tat(−) and Tat(+) mice. Data are shown as box and dot plots with diamonds representing single animal values. The box represents the interquartile range, with the line representing the median and the square representing the mean. Whiskers show the SD. Data were analysed by 1-way ANOVA (the Bonferroni post hoc test). The *P* value in the upper left corner represents the overall group difference. (C) Percentage of area with positive immunoreactivity to GFAP in Tat(−) and Tat(+) mice. Data are shown as box and dot plots with diamonds representing single animal values. The box represents the interquartile range, with the line representing the median and the square representing the mean. Whiskers show the SD. Data were analysed by 1-way ANOVA (not significant). The *P* value in the upper left corner represents the overall group difference. ANOVA, analysis of variance; DOX, doxycycline; GFAP, glial fibrillary acidic protein.

A significant increase in the number of microglial profiles was measured in Tat(+) mice as compared to Tat(−) mice after both 8 days (*P* < 0.001) and 6 weeks (*P* = 0.007; Fig. 4A, B) of DOX treatment. By contrast, no difference in immunoreactivity for astrocytes was present between control and Tat-expressing mice (*P* = 0.24; Fig. 4A, C).

### 3.5. Tat(+) mice display mechanical, but not thermal, hypersensitivity

**Figure 5.**
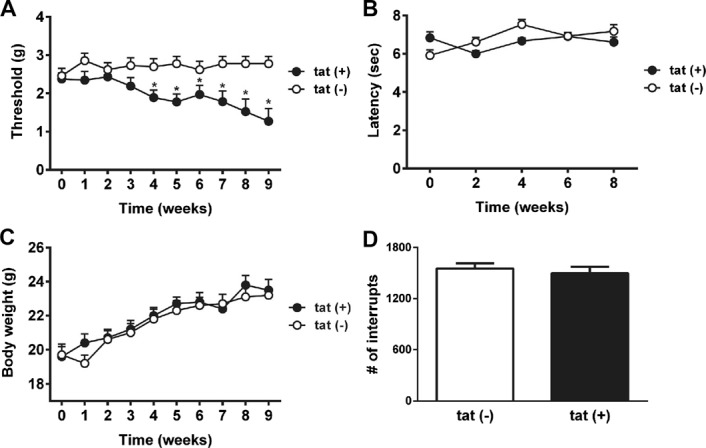
Behavioural outcomes in Tat(−) and Tat(+) mice. (A) Paw withdrawal thresholds (PWTs) over time—von-Frey test. (B) Paw withdrawal latencies (PWLs) over time—Hargreaves test. (C) Body weight over time. (D) Locomotor activity (number of infrared beam interruptions). Data shown as mean ± SEM (n = 10 mice per group). Data were analysed by a 2-way repeated-measures ANOVA (Sidak) for multiple group comparison and by an unpaired Student *t* test for comparison of 2 groups. *P < 0.05. ANOVA, analysis of variance.

Mechanical and thermal withdrawal thresholds were comparable between Tat(+) and Tat(−) mice before DOX diet exposure (*P* > 0.05; Fig. 5A, B). Over the duration of the study, no significant difference in body weight gain between genotypes was detected (genotype [F(1, 9) = 0.38; *P* > 0.05]; time [F(9, 81) = 67.95; *P* < 0.001]; genotype × time interaction [F(9, 81) = 1.67; *P* > 0.05]; Fig. 5C). Mechanical paw withdrawal thresholds showed significant main effects of genotype (F(1, 9) = 8.85; *P* < 0.05), time (F(9, 81) = 2.35; *P* < 0.05), and genotype × time interaction (F(9, 81) = 4.37; *P* < 0.001). Significantly reduced mechanical paw withdrawal thresholds were present after 4 weeks of DOX exposure and continued progressively until the end of study (*P* < 0.05; Fig. 5A). By contrast, thermal withdrawal latencies (PWLs) showed no significant main effect of genotype (F(1, 9) = 1.01; *P* > 0.05) and time (F(4, 36) = 4.38; *P* > 0.05). Although an overall significant genotype × time interaction was present (F(4, 36) = 6.95; *P* < 0.001), differences in PWLs between Tat(+) and Tat(−) mice never reached significance at any of the time points measured (*P* > 0.05; Fig. 5B). No significant difference in spontaneous motor activity was measured between Tat(+) and Tat(−) mice (*P* > 0.05; Fig. 5D).

## 4. Discussion

Here, we investigated the potential role of the viral protein Tat in HIV-SN pathogenesis. We demonstrated reduced epidermal nerve fibre density, a marker for neuropathy, in Tat(+) mice. Tat(+) animals also displayed a progressive mechanical hypersensitivity after long-term Tat exposure.

To the best of our knowledge, this is the first time that the effect of Tat on epidermal nerve fibre density has been shown. Quantification of intraepidermal nerve fibres is a commonly used, validated, diagnostic tool for small fibre neuropathies.^40,41^ Importantly, loss of epidermal nerve fibres has been shown in patients with HIV-SN^56^ and simian immunodeficiency virus (SIV)-infected macaques.^39^ Tat mRNA is detectable within the peripheral nervous system including the lumbar DRG and skin since satellite cells and Schwann cells can express GFAP.^30,48^ There is also evidence that Tat is expressed by enteric glia in Tat(+) mice.^52^ We showed significant *tat* mRNA expression in the lumbar DRG and skin at 8 days of DOX exposure, and, although not significant, a trend (*P* = 0.052) towards an increase in the lumbar spinal cord. Relatively slow onset of *tat* expression in the spinal cord vs other CNS regions has been shown previously,^19^ and may depend on spatial and temporal differences in Tat expression, regulated by regional and ontogenetic changes in GFAP-dependent rtTA promoter expression. Interestingly, Tat mRNA levels were significantly increased after 8 days of Tat expression, whereas the effect on epidermal nerve fibres was only measurable after 6 weeks. This suggests that the length of Tat exposure is an important factor for Tat-induced neurotoxicity. It has been suggested that gp120, the viral protein primarily studied in relation to HIV-SN, plays an important role in the early stages of HIV-SN.^61^ In rodent models of gp120-induced neuropathy, nerve fibre loss is measurable after 14 days of gp120 exposure.^70,71^ The delayed loss of epidermal nerve fibres shown here might indicate a role of Tat in the maintenance of HIV-SN at later stages of the HIV infection. Interestingly, secretion of Tat is unaffected by antiretroviral treatment.^47^ Soluble Tat, sustained by a small Tat reservoir in infected cells, can be present throughout chronic HIV infection, despite low viral loads, which could contribute to the high prevalence of HIV-SN in the cART era.^47^

As activation of immune cells has been shown in primate models of HIV infection and in gp120-induced rodent models of HIV-SN^23,37,70,71,81^ and has been linked to Tat-induced neurotoxicity in vitro^16,43,62,66,80^ and in vivo,^8,24,36,46^ we assessed immune cells in the DRG and spinal cord. No macrophage or satellite cell activation was measured in Tat-expressing mice. The lack of immune cell activation in the DRG further supports the difference between gp120- and Tat-induced neuropathy. In particular, macrophage infiltration and satellite glial cell activation has been shown in rats exposed to gp120.^70,71^ Macrophage activation has also been shown in the DRG in SIV-infected macaques.^9,37,39^ The SIV models reporting macrophage activation are predominantly fast-progressing models, characterised by high viral loads, and therefore only inform about DRG pathogenesis at early stages of untreated infection. It may also be that macrophage activation occurs at a time point not investigated here. A temporal study would be therefore crucial for future experiments. In contrast to the discrepancy in DRG pathogenesis, spinal microgliosis observed in Tat(+) mice is also present in gp120-induced neuropathy.^70,71^ Activation of spinal microglia has been described in other neuropathy models characterised by epidermal nerve fibre loss including nerve injury–induced and diabetic neuropathy and is believed to play an important role in the maintenance of neurological complications such as neuropathic pain.^13,22,77^ Here, spinal astrocyte activation was not shown in spinal cords of Tat(+) mice. This is in contrast to a previously reported Tat transgenic model with a higher transgene copy number (3–10 copies) that generally results in a more severe neuropathology with an earlier onset.^36^ By contrast, the mice used in this study only express only a single copy of the *tat* gene.^8^

We also demonstrated the development of mechanical hypersensitivity in Tat(+) mice. Notably, mechanical hypersensitivity was measureable after 4 weeks of DOX exposure, suggesting that the length of Tat exposure is a key factor in Tat-induced SN. Mechanical hypersensitivity without thermal hypersensitivity has also been shown in gp120-induced neuropathy.^70,71^ Interestingly, mechanical hypersensitivity develops after days of gp120 exposure,^70,71^ whereas HIV Tat results in mechanical hypersensitivity weeks after exposure. Whether this is indicative of a role of gp120 in the early phases of HIV infection and of HIV Tat in the maintenance of HIV-SN has still to be determined. It may be that the difference in the time courses is due to methodological discrepancies. Although gp120 was directly applied to the sciatic nerve, expression of Tat in the transgenic mouse causes a more generalized distribution because of production by astroglia and possibly by peripheral satellite and Schwann cells. It is noteworthy that hypersensitivity is not a common clinical sign reported by patients with HIV-SN; indeed, sensory loss is much more prominent.^50,54^ This phenomenon has also been reported in diabetic neuropathy and chemotherapy-induced neuropathy in animal models, whereas patients commonly suffer from sensory loss.^45,51,63,67,68,77^ However, the data presented here are in line with the gp120 literature.^29,70,72^ The argument is that the animal models are probably looking at the initial pathogenic phase where sensory gain may well be the phenotype, whereas the clinical studies generally look at patients with well-established neuropathies. The discrepancy between clinical data and experimental findings in animals may be due to differences in the time point when sensory losses are assessed.^59^ Animal studies investigate mainly the early stages of neuropathy, whereas clinical studies look at patients with longstanding HIV infections, although a recent study showed HIV peripheral neuropathy only months after infection.^73^ Sensory loss might be demonstrated if animal models were profiled for extended periods, as was shown in a model of diabetic neuropathy.^10^ It also has been suggested that damage to nerve fibre endings can trigger hyperexcitable nociceptive fibres resulting in spontaneous nerve firing and hypersensitivity.^15^ Reporting biases in preclinical trials that exclude animals not showing hypersensitivity might mask the detection of sensory losses in animal models.^28^ Importantly, no mice were excluded from behavioural testing in our studies. It is also noteworthy that Tat expression in these studies had no effect on locomotor activity, suggesting that motor function is not affected by Tat at this length of exposure, which is also in contrast to the Kim et al.^36^ model. Motor behavior in the present model does develop by 3 months of DOX exposure.^24^ Notably, patients with HIV often report chronic pain states and comorbid complications.^34,54^ In animals exposed to gp120, a reduction of spontaneous exploratory behaviour in the open-field paradigm, an ethological behaviour believed to measure more complex comorbidities of neuropathic pain has been shown.^70,71^ Another exploratory behaviour recently discussed as a useful tool to investigate neuropathic pain is the burrowing paradigm.^78^ It would be of interest to further investigate the effect of Tat expression on these more complex, nonevoked behaviours.

Neither the number of macrophages or GFAP-immunoreactive cells in the DRG, nor the number of astrocytes in the dorsal spinal cord, seemed to correlate with the hypersensitivity behaviours. However, increases in the number of microglia in the dorsal spinal cord did coincide with heightened pain sensitivity. It is possible that Tat expression is directly excitotoxic causing epidermal nerve fibre loss^25,57^ and perhaps, in subsequent hypersensitivity. It is also possible that peripheral nerve damage can activate glia within the spinal cord, trigger the release of proinflammatory cytokines, and increase the firing rate of neurons responsible for hypersensitivity, as has been proposed in other model systems.^31^ Future experiments would be crucial to further characterise this model.

Although the identity of the cells expressing Tat was not shown, qRT-PCR or dPCR demonstrated expression in all expected tissues. It is likely that Tat is expressed centrally in subsets of astrocytes, and also peripherally in subsets of Schwann cells and satellite cells. Identifying the cellular sites of Tat expression would be an important next step.

Another notable point is the use of female animals in this study, whereas rodent studies investigating gp120-induced neuropathy were predominantly performed in male rats.^70,71^ As previously mentioned, 52% of people who are currently living with HIV are women^1^ and no significant difference between HIV patients with and without HIV-SN in terms of sex has been shown.^54,64^ However, it has been recently suggested that microglia are involved in the development of mechanical pain hypersensitivity in male, but not in female mice.^65^ Therefore, further studies in male Tat(+) mice are warranted to verify whether Tat expression elicits similar differences in immune cell responsiveness across sexes. To further differentiate mechanisms linked to Tat as compared to gp120, additional immunostaining for markers that are upregulated in gp120-induced neuropathy might be of interest.^71^ It may also be important to measure proinflammatory cytokines in Tat(+) mice. HIV-1 Tat acts to recruit monocyte-derived cells and promotes chemokine and/or cytokine expression,^2,38^ partly through NF-κB activation.^27^ Moreover, Tat interacts with opiates, frequently used for pain syndromes, to activate macrophages/microglia and potentiate cytokine expression in vitro.^7,8,16,18,20^ In support, the phosphodiesterase inhibitor, ibudilast, quiets human and murine microglia in vitro and attenuates Tat-facilitated proinflammatory cascades.^35^ The extent to which anti-inflammatory adjunctive therapies may ameliorate peripheral neuropathic pain in this model is not known; however, COX-1/COX-2 inhibition attenuates affective behavioural deficits associated with cortical microglial activation in transgenic Tat mice.^53^ Indeed, single-nucleotide polymorphisms in genes-encoding purinergic receptors (*P2X7R* and *P2X4R*) and TNF-α haplotypes predicted HIV-SN in African populations.^21,26,69^ Future investigations may aim to ameliorate Tat-associated peripheral neuropathy, in part, through pursuit of anti-inflammatory therapeutics.

A crucial future step would be to provide a link between experimental work and the clinic. In mice, overexpression of Tat results in CNS damage and cognitive deficits.^24,36^ In patients, it has been shown that high levels of Tat antibodies in the cerebral spinal fluid inversely correlate with dementia severity, suggesting that high antibody levels protect against Tat toxicity by sequestering Tat function.^3^ However, the presence of antibodies against Tat only serves as a proxy estimate.^3,6,58^ Although direct assessments of Tat have occasionally been made,^32^ routine measurements would be extremely useful to more accurately assess the role of Tat in the neuropathogenesis of HIV-SN.

Overall, we demonstrated epidermal nerve fibre loss in Tat(+) mice, a marker of HIV-SN. We also showed mechanical hypersensitivity after long-term Tat expression. Importantly, the data suggest an underlying mechanism distinguishable from gp120-induced neuropathy. This emphasises the potential importance of the murine model described here to better understand the underlying mechanisms of HIV-SN pathogenesis and ultimately improve patient care.

## Disclosures

The authors have no conflict of interest to declare.

This study was funded by the NIAA-BJA/RCoA (R.W., T.P., and A.S.C.R.), the Joint Research Committee of the Chelsea and Westminster NHS Trust (R.W., T.P., and A.S.C.R.), R01 CA206028 to M.I. Damaj, and NIDA (K99 DA039791 to J.J.P.; K02 DA027374 to K.F.H.; and R01 DA034231 to P.E.K. and K.F.H.).

## Appendix A. Supplemental digital content

Supplemental digital content associated with this article can be found online at http://links.lww.com/PR9/A17.
